# ﻿The genus *Inversotyphlus* Strasser, 1962, stat. nov. and *Inversotyphlusammirandus* sp. nov., a new bizarre, highly modified troglobiotic millipede (Diplopoda, Julida, Julidae) from Albania, Balkan Peninsula

**DOI:** 10.3897/zookeys.1184.113498

**Published:** 2023-11-14

**Authors:** Dragan Antić, Nesrine Akkari

**Affiliations:** 1 University of Belgrade–Faculty of Biology, Institute of Zoology, Center for Biospeleology, Studentski trg 16, 11000 Belgrade, Serbia University of Belgrade Belgrade Serbia; 2 Serbian Biospeleological Society, Trg Dositeja Obradovića 2, 21000 Novi Sad, Serbia Serbian Biospeleological Society Novi Sad Serbia; 3 3rd Zoological Department, Natural History Museum Vienna, Burgring 7, 1010 Vienna, Austria Natural History Museum Vienna Vienna Austria

**Keywords:** Cave, Dinarides, hygropetric, millipede, modified mouthparts, taxonomy, Typhloiulini, *Typhloiulus*

## Abstract

*Inversotyphlus* Strasser, 1962, **stat. nov.** is raised to the genus level and a new hydrophilous species, *Inversotyphlusammirandus***sp. nov.**, is described from the second deepest pit in Albania. This species is characterized by a highly modified head and body for a presumably semiaquatic or hygropetric life and filtering diet. It is by far the most bizarrely modified cave-dwelling julid known. The new species is diagnosed, described in detail, and richly illustrated. Besides *I.ammirandus***sp. nov.**, the genus *Inversotyphlus***stat. nov.** includes six species: *I.clavatus* (Antić, 2018), **comb. nov.**, *I.edentulus* (Attems, 1951), **comb. nov.**, *I.gellianae* (Makarov & Rađa, 2006), **comb. nov.**, *I.gracilis* (Antić, 2018), **comb. nov.**, *I.lobifer* (Attems, 1951), **comb. nov.**, and *I.opisthonodus* (Antić, 2018) **comb. nov.** The subgenus Attemsotyphlus**syn. nov.** is here considered as a junior subjective synonym of the genus *Inversotyphlus***stat. nov.** Notes are given on the habitat of *I.ammirandus***sp. nov.**, the taxonomy of the tribe Typhloiulini and the genus *Inversotyphlus***stat. nov.**, and adaptive modifications of the mouthparts.

## ﻿Introduction

The Balkan Peninsula is known as one of the richest regions in the world for subterranean fauna. The area is inhabited by numerous highly adapted troglomorphic hypogean species. This is especially true for the Dinaric part of the Balkans, which is extremely rich in aquatic and terrestrial cave taxa and is considered a global hotspot of subterranean biodiversity (Culver and Sket 2000). Despite almost two centuries of intensive speleobiological research in this region, which makes it the most investigated region in the world, some parts of the Dinarides and the Balkans in general are still very poorly studied. One of the least speleobiologically researched areas is certainly within the territory of Albania. Just over 50 species in the class Diplopoda have been recorded from the country, and only a handful of them are known from subterranean habitats (Mauriès et al. 1997; Stoev 2001; Stoev and Enghoff 2008; Antić et al. 2015).

The best-known group of subterranean julids in the Balkan Peninsula is undoubtedly the controversial tribe Typhloiulini, which has several genera. Among them, the genus *Typhloiulus* Latzel, 1884 (see Latzel 1884) has the widest distribution and the largest number of species. Interestingly, this genus includes three highly modified, hydrophilous cave species with modified mouthparts and a presumably filtering diet: *T.balcanicus* Antić, 2017, *T.edentulus* Attems, 1951, and *T.serbani* (Ceuca, 1956) (see Attems 1951, 1959; Ceuca 1956; Antić et al. 2017). Traditionally, *Typhloiulus* includes several subgenera, some of which are well defined (Vagalinski et al. 2015; Antić et al. 2018). One of these well-defined lineages of Typhloiulus is the Dinaric subgenus Inversotyphlus Strasser, 1962 (see Strasser 1962). According to Antić et al. (2018), this group includes five or six species known from the caves of the coastal areas and islands of the central and southern Dinarides.

Thanks to the collecting efforts of Croatian speleobiologists, we have the opportunity to describe here a new Dinaric troglobiotic and highly modified species of *Inversotyphlus*, from a deep pit in Albania. This is by far the most bizarre hydrophilous cave-dwelling millipede discovered to date. In addition, here we elevate *Inversotyphlus* stat. nov. to full generic rank.

## ﻿Material and methods

**Figure 1.**
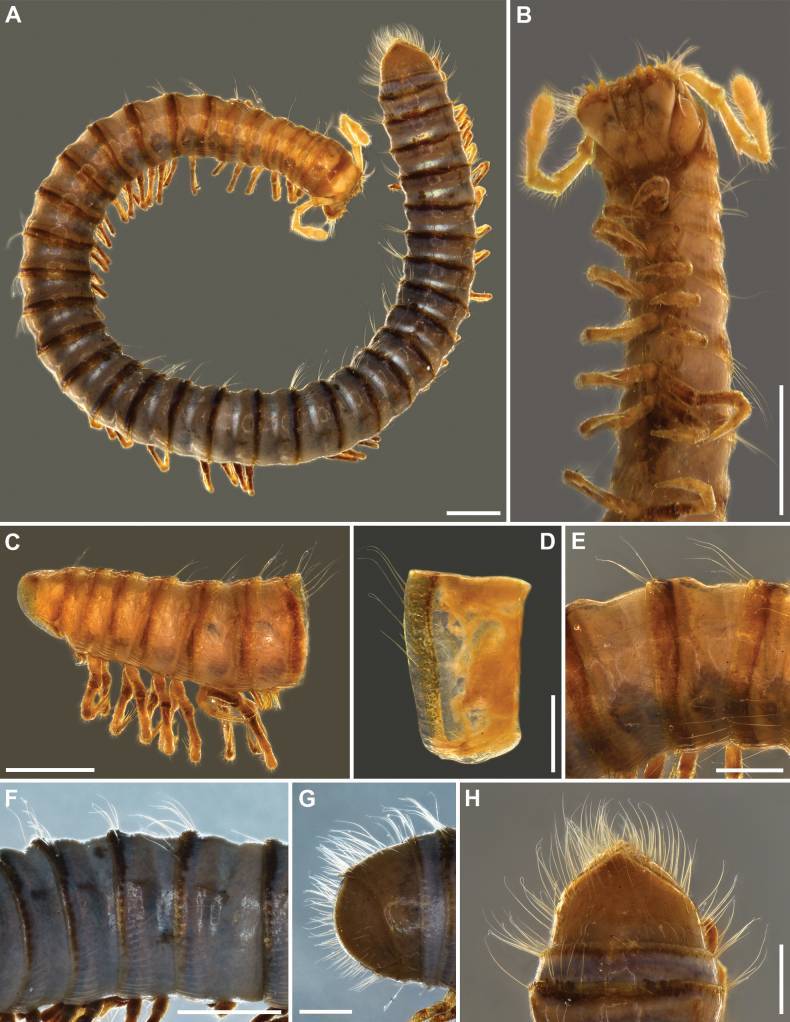
*Inversotyphlusammirandus* sp. nov., holotype ♂ (NHMW MY10380), habitus **A** whole animal, dorsal view **B** anterior part of body, ventral, somewhat lateral view **C** body segments 1–7, with leg-pair 1, walking legs and gonopods, lateral view **D** right half of pleurotergum 7, lateral view **E** body rings 7–9, dorsolateral view **F** mid-body rings, lateral view **G** end of body, lateral view **H** end of body, dorsal view. Scale bars: 1 mm (**A–C, F**); 0.5 mm (**D, E, G, H**).

**Figure 2.**
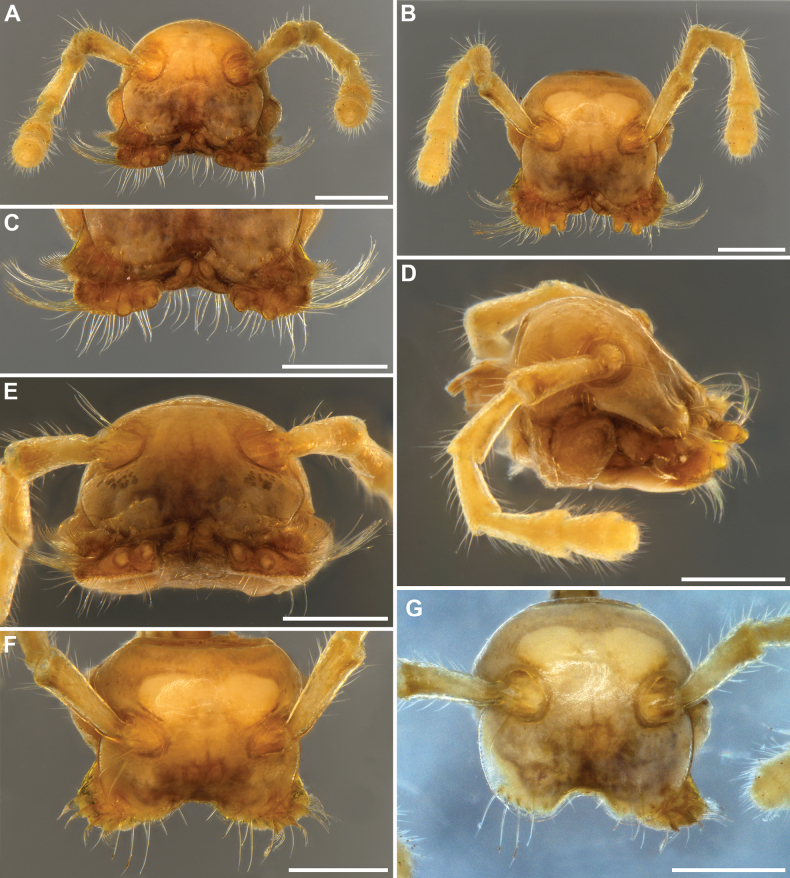
*Inversotyphlusammirandus* sp. nov., holotype ♂ (NHMW MY10380), head **A** anterodorsal view **B** dorsal view **C** anterior part of head, anterodorsal view **D** lateral view **E** anterior view **F** head without gnathochilarium, dorsal view **G** head without gnathochilarium and right mandible, dorsal view. Scale bars: 0.5 mm.

**Figure 3.**
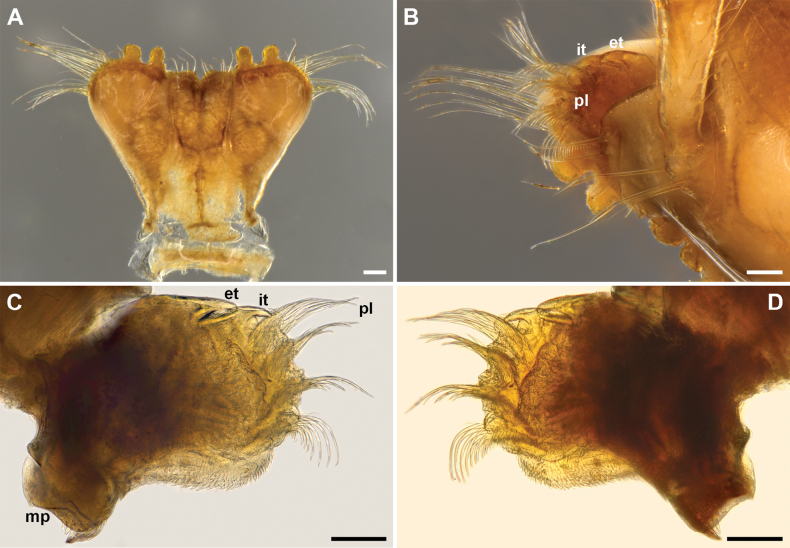
*Inversotyphlusammirandus* sp. nov., holotype ♂ (NHMW MY10380), mouthparts **A** gnathochilarium, ventral view **B** right anterior part of head, showing antennomere 1 and 2, labral lobe, mandible and stipital bundle **C** right mandible, ventral view **D** right mandible, dorsal view. Abbreviations (mandible): **et** external tooth, **it** internal tooth, **mp** molar plate, **pl** pectinate lamellae. Scale bars: 0.1 mm.

**Figure 4.**
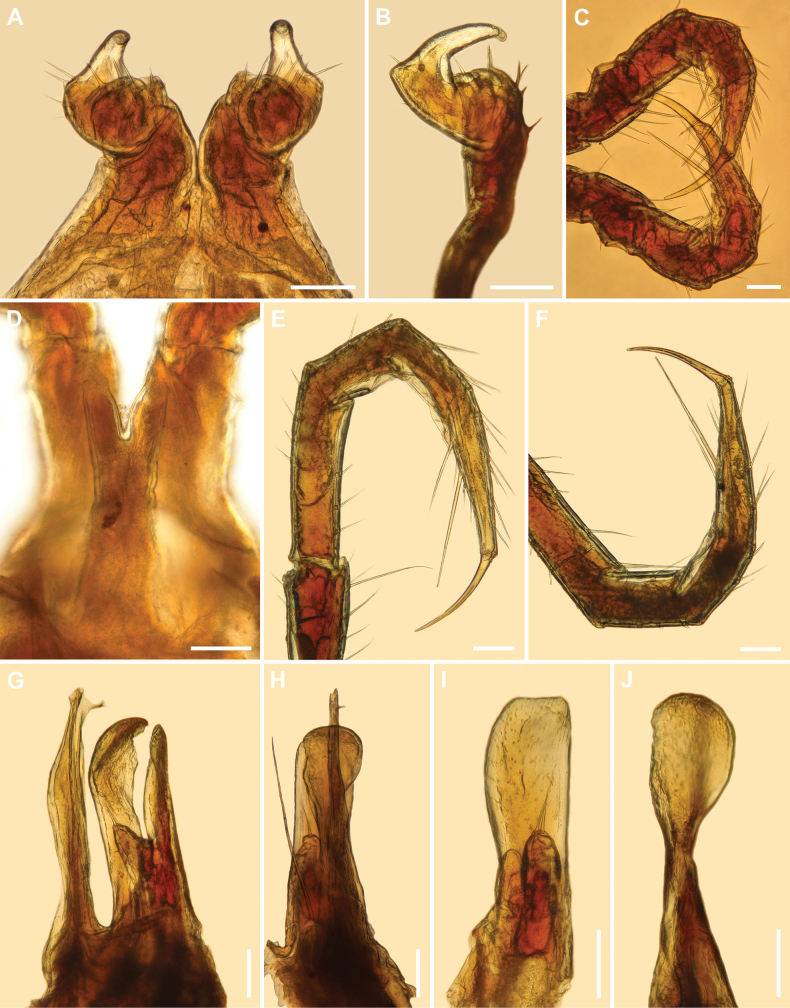
*Inversotyphlusammirandus* sp. nov., holotype ♂ (NHMW MY10380) **A** leg-pair 1, anterior view **B** left leg 1, lateral view **C** telopodites of leg-pair 2, posterior view **D** penis and coxae of leg-pair 2, posterior view **E** left telopodite 10, anterior view **F** right mid-body telopodite, anterior view **G** right gonopods, mesal view **H** left gonopods, posterior view **I** right promere, posterior view **J** right mesomere, anterior view. Scale bars: 0.1 mm.

The holotype, preserved in 70% ethanol, was examined with a Nikon SMZ745T (University of Belgrade – Faculty of Biology, Serbia; IZB) and a Nikon SMZ18 (Naturhistorisches Museum Wien, Austria; NHMW) binocular stereomicroscopes. All taxonomically important structures were dissected and mounted in glycerine as temporary microscope preparations and observed with a Carl Zeiss Axioskop 40 microscope (IZB) and a Nikon Eclipse Ni microscope (NHMW). The measurements were taken with a Carl Zeiss Stemi 2000-c binocular stereomicroscope with an AxioCam MRc camera (IZB) using software for measuring. Photographs of habitus and external characters were taken using a Nikon DS-Fi2 camera with a Nikon DS-L3 camera controller attached to a Nikon SMZ1270 binocular stereomicroscope (IZB; Figs 1F, G, 2G) and a Nikon DS-Ri-2 camera mounted on a Nikon SMZ25 binocular stereo microscope using NIS-Elements Microscope Imaging Software with an Extended Depth of Focus (EDF) patch (NHMW; Figs 1A–E, H, 2A–F, 3). Pictures of mandible, gonopods, legs, and penis were made with a Canon PowerShot A80 digital camera connected to a Carl Zeiss Axioskop 40 microscope (IZB; Figs 3D, 4B, C, E–G, I, J, 6) and a DS-Ri-2 camera mounted on a Nikon Eclipse Ni microscope using NIS-Elements Microscope Imaging Software with an EDF patch (NHMW; Figs 3A–C, 4A, D, H). Line drawings of the gonopods, first pair of legs, and penis were made using tracing paper placed on a computer monitor displaying images of those structures made with a Canon PowerShot A80 digital camera connected to a Carl Zeiss Axioskop 40 microscope (IZB). The images were processed and assembled in Adobe Photoshop CS6.

The abbreviations of the gonopodal and mandibular structures are explained directly in the text and in the figure legends. The terminology used to denote the different parts of gonopods follows Antić et al. (2018).

The holotype male is deposited in the Naturhistorisches Museum Wien, Vienna, Austria (**NHMW**). Specimens of taxa described earlier and mentioned below (Fig. 6) are deposited in the Institute of Zoology, University of Belgrade — Faculty of Biology, Belgrade, Serbia (**IZB**) and in the Natural History Museum Split, Croatia (**NHMSC**).

## ﻿Results

### ﻿Taxonomy

**Class Diplopoda de Blainville in Gervais, 1844**

**Order Julida Brandt, 1833**

**Family Julidae Leach, 1814**

#### ﻿Genus Inversotyphlus

Taxon classificationAnimaliaJulidaJulidae

Strasser, 1962 stat. nov.

1859161C-423C-5E33-BAD4-6F9B7F2EFEC4

Inversotyphlus Strasser, 1962: 45 (as subgenus of Typhloiulus Latzel, 1884).
Attemsotyphlus Strasser, 1962: 47 (as subgenus of Typhloiulus Latzel, 1884; type species Typhloiulusedentulus Attems, 1951 by monotypy); syn. nov.

##### Type species.

*Typhloiuluslobifer* Attems, 1951 by monotypy.

##### Diagnosis.

Blind and mostly depigmented species of the julid tribe Typhloiulini, with a pair of frontal setae, promere with flagellum, mesomere free, opisthomere slender. Differs from other members of the tribe Typhloiulini in the absence of an opisthomesomeral lamella, the presence of an opisthomeral velum ending with a thin process carrying a few fimbriae, presence of a poorly developed but noticeable anterior lamella of the opisthomere, and the simple, more or less blunt apex of the solenomere in combination of a hook-shaped leg-pair 1 and a slender penis with two long distal lobes.

###### Included species

*Inversotyphlusclavatus* (Antić, 2018), comb. nov. (ex *Typhloiulus*).

*Inversotyphlusedentulus* (Attems, 1951), comb. nov. (ex Typhloiulus (Attemsotyphlus)).

*Inversotyphlusgellianae* (Makarov & Rađa, 2006), comb. nov. (ex *Typhloiulus*).

*Inversotyphlusgracilis* (Antić, 2018), comb. nov. (ex *Typhloiulus*).

*Inversotyphluslobifer* (Attems, 1951), comb. nov. (ex *Typhloiulus*).

*Inversotyphlusopisthonodus* (Antić, 2018), comb. nov. (ex *Typhloiulus*).

###### New species

#### ﻿ Inversotyphlus ammirandus sp. nov.

Taxon classificationAnimaliaJulidaJulidae

92000BE4-0751-58B5-BAD8-88FBEC86BD8C

https://zoobank.org/BCE36811-7013-4F70-B8DB-4CED5E055FD2

**Figure 5.**
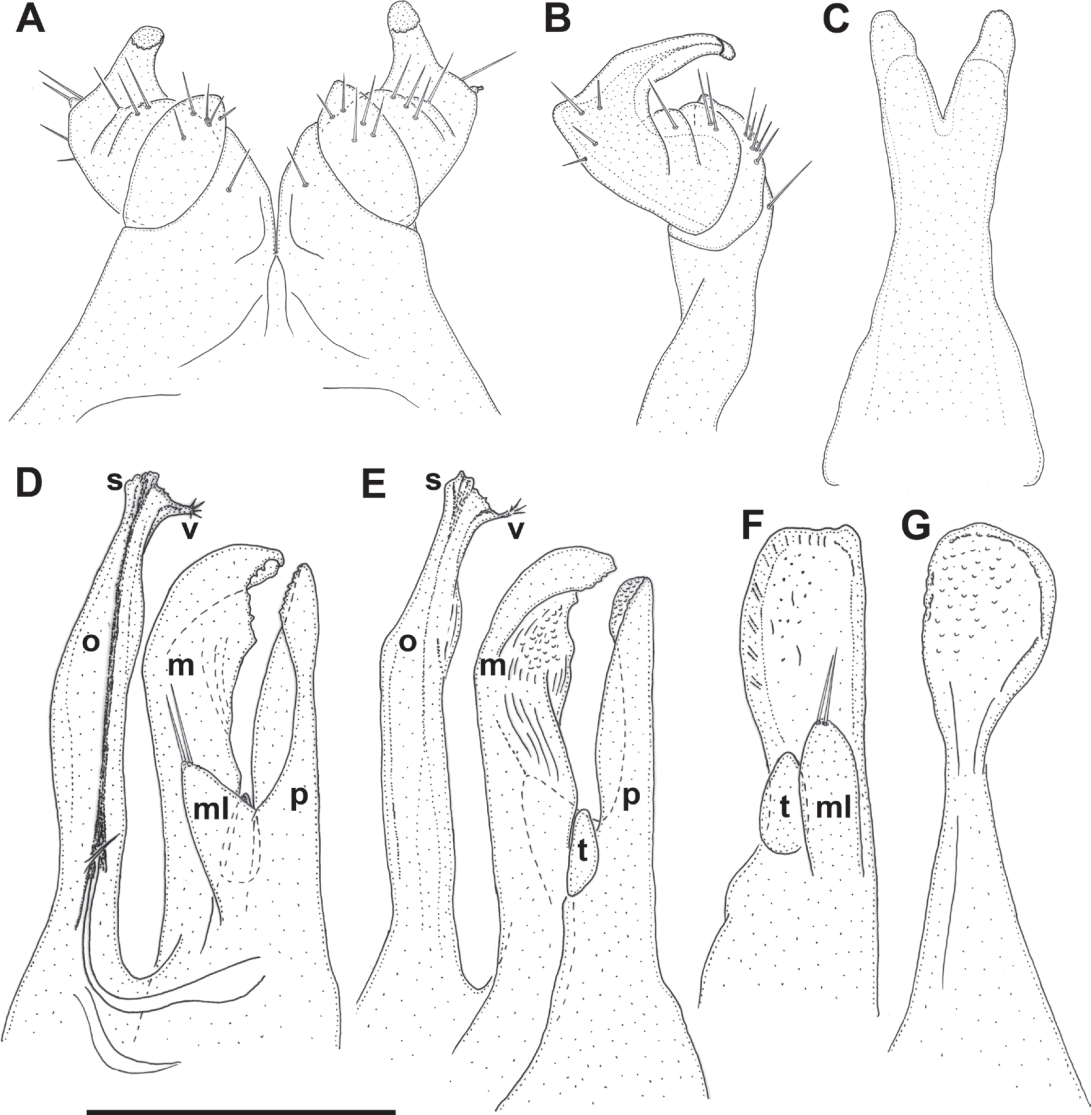
*Inversotyphlusammirandus* sp. nov., holotype ♂ (NHMW MY10380) **A** leg-pair 1, anterior view **B** left leg 1, lateral view **C** penis, posterior view **D** right gonopods, mesal view **E** left gonopods, lateral view **F** right promere, posterior view **G** right mesomere, anterior view. Abbreviations: **m** mesomere, **ml** mesal lobe, **o** opisthomere, **p** promere, **s** solenomere, **t** telopodite, **v** velum. Scale bars: 0.3 mm.

**Figure 6.**
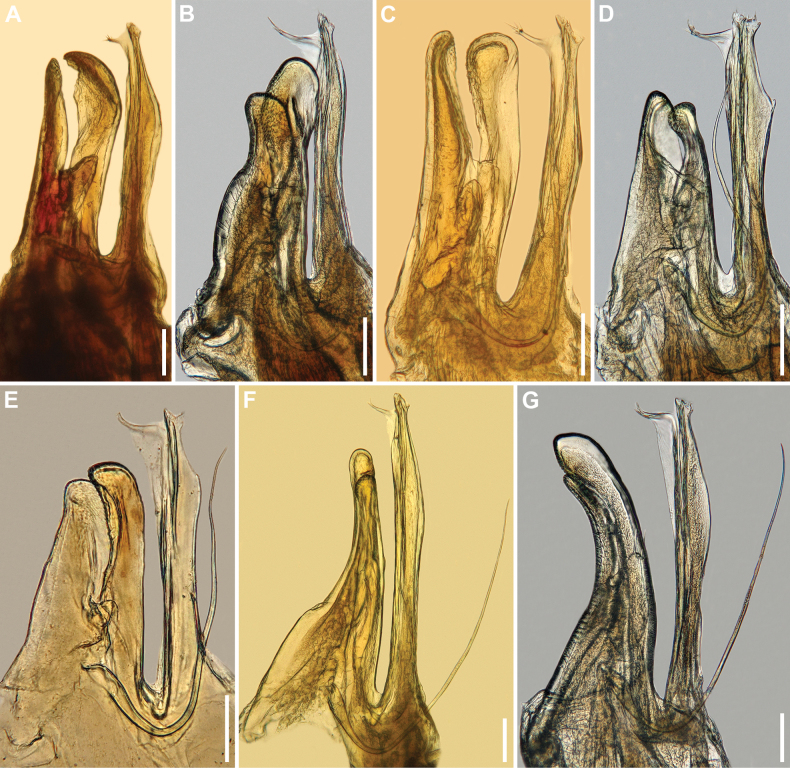
Gonopods of the members of the genus *Inversotyphlus* Strasser, 1962 stat. nov., mesal views **A***I.ammirandus* sp. nov., holotype ♂ (NHMW MY10380), right gonopods (flipped) **B***I.clavatus* (Antić, 2018), comb. nov., holotype ♂ (NHMSC), left gonopods **C***I.edentulus* (Attems, 1951), comb. nov., ♂ from Njegoš Cave (IZB), left gonopods **D***I.opisthonodus* (Antić, 2018), comb. nov., holotype ♂ (NHMSC), left gonopods **E***I.gellianae* (Makarov & Rađa, 2006), comb. nov., holotype ♂ (IZB), left gonopods **F***I.gracilis* (Antić, 2018), comb. nov., paratype ♂ (IZB), left gonopods **G***I.lobifer* (Attems, 1951), comb. nov., ♂ from bitumen mine Minjera, island of Brač (IZB), left gonopods. **B, D–G** modified after Antić et al. (2018). Scale bars: 0.1 mm.

Figs 1 , 2 , 3 , 4 , 5 , 6A

##### Diagnosis.

The new species is distinguished from all congeners and other members of Typhloiulini by its highly modified head, which is characterised by having two labral lobes with a wide incision between to accommodate the lingual palps, a unique gnathochilarium that is distally very wide, bearing a distolateral bundle of long setae on the stipites and distal (anterior) rows of long setae on both the stipites and lamellae linguales (such structures are absent in all congeners). In addition, the body rings are strongly vaulted and bear very long metazonal setae; the tarsal claws are very long.

Concerning the gonopods, the new species is characterised by a slightly higher mesomere compared to the promere (vs mesomere lower than promere in *I.opisthonodus* comb. nov. and *I.gracilis* comb. nov., or mesomere much higher than promere in *I.lobifer* comb. nov. and *I.clavatus* comb. nov., or mesomere and promere of equal height in *I.edentulus* comb. nov.). Except in the new species, a mesomere slightly higher than the promere is found only in *I.gellianae* comb. nov., but *I.ammirandus* sp. nov. differs from this and all other members of *Inversotyphlus* stat. nov. in the presence of a spoon-shaped mesomere. Further differences in the gonopods can be seen in Fig. 6.

##### Name.

From the Latin *ammirandus* (= wonderful, admirable, astonishing, remarkable, extraordinary), reflecting the impressive appearance of this bizarre creature. Moreover, this species name is dedicated to all speleologists and speleobiologists around the world who risk their lives exploring deep caves, which makes them admirable too. Adjective in masculine gender.

##### Material examined.

***Holotype*** ♂ (NHMW MY10380), Albania, Shkodër County, Malësi e Madhe Municipality, Bogë village, Prokletije Mountain range, Radohimës (Radohinës) mountain group, Cave Ru, –460 m, 8 September 2013, T. Čuković leg.

##### Description.

***Number of body rings and size***: 30 mm long, vertical diameter of largest body ring 1.5 mm, body with 36 podous rings + 1 apodous ring + telson.

***Colouration***: after a decade in ethanol, generally greyish brown (Fig. 1). Anterior part of body and telson rather yellowish brown (Fig. 1A, B). Antennae and forehead yellowish; legs brownish. Metazonae with dark-brown posterior rings.

***Head***: highly modified (Figs 2, 3), without ommatidia, with a pair of frontal setae (Fig. 2A, E; both fallen off). Labrum considerably shorter than gnathochilarium, with two rounded lobes with a wide inscision between lobes to accomodate lingual palps; no labral teeth; 3+3 short labral and 5+5 long supralabral setae (Figs 2, 3B). Gnathochilarium (Fig. 3A) subtrapezoidal, with a very wide distal part; stipites strongly developed, with rounded distolateral margins and characteristic long distal setae in a transverse row and distolateral bundles; lamellae linguales rectangular, with a transverse row of long distal setae, a group of somewhat shorter proximal setae, and a longitudinal row of lateral setae, lingual palps directed dorsad; promentum very short, deltoid. Mandibles (Figs 2A–F, 3B–D) highly modified, dorsoventrally strongly flattened, external (**et**) and internal tooth (**it**) reduced, four pectinate lamellae (pl) hypetrophied, with very long outher pectinate teeth, molar plate (**mp**) reduced. Antennae 2.30 mm long, their length ca 150% of vertical diameter of widest body ring. Length of antennomeres I–VIII (in mm): 0.17 (I), 0.57 (II), 0.40 (III), 0.35 (IV), 0.43 (V), 0.27 (VI), 0.07 (VII) and 0.04 (VIII). Length/width ratio of antennomeres I–VII: 1 (I), 2.8 (II), 2 (III), 2 (IV), 1.7 (V), 1 (VI) and 0.5 (VII). Antennomere I with a few very long anterior setae (Figs 2E, 3B); antennomeres V and VI each with a terminal corolla of large sensilla basiconica bacilliformia; antennomere VII with a terminal corolla of small sensilla basiconica bacilliformia.

***Body rings***: body in general moniliform (Fig. 1A). Metazonae strongly vaulted compared to prozonae (Fig. 1A, C, E, F). Entire metazonal area with longitudinal striations, striae more numerous and denser below ozopore and rarer and less conspicuous above ozopore (Fig. 1E). Length of metazonal setae ca 45% of vertical diameter of rings, ca 40 per ring (Fig. 1E, F). Posterior margin of metazona thickened dorsolaterally (Fig. 1F). Ozopores behind pro-metazonal suture at ca ½ of metazonal length (Fig. 1F).

***Pleurotergum 7***: ventral margin with very low, poorly developed lobe (Fig. 1C, D).

***Telson***: pre-anal ring densely setose, with a short, acuminate epiproct (Fig. 1G, H). Paraprocts rounded, each with ca 30–35 long setae over entire surface. Hypoproct in form of symetrical subtriangle, covered with ca 15 long setae, without any modifications.

***Legs***: leg-pair 1 modified, hook-shaped, with three complete podomeres; coxa with one seta; prefemur with five setae; femur, postfemur, and tibiotarsus coalesced, with indications of segmentation; femur with two or three setae; postfemur with one seta; tibiotarsal part with a small distal lobe and four setae; tip slightly tuberculate (Figs 1C, 4A, B, 5A, B). Anterior walking legs with an adhesive pad on tibia (Fig. 4C, E), most pronounced in leg-pair 2 (Fig. 4C), gradually disappearing in posterior direction, completely disappearing around mid-body (Fig. 4F). Length of mid-body legs ca 1.45 times as long as mid-body vertical diameter; tarsus ca 2 times as long as tibia with very long, spinelike ventral seta; apical claws strongly developed, very long, ca 60% of tarsus length (Fig. 4F); no accesory claw.

***Penis***: bilobed. Lobes prominent, elongated (Figs 4D, 5C).

***Gonopods***: *in situ* protruding from gonopodal sinus (Fig. 1B, C). Promere (**p**) and mesomere (**m**) surpassed by opisthomere (**o**) (Figs 4G, H, 5D, E, 6A). Promere (Figs 4G, I, 5D–F, 6A) somewhat shorter than mesomere; subrectangular in anterior and posterior views; sides almost parallel, mesal margin straight, lateral margin slightly convex; distally microsquamose; mesal lobe (**ml**) well developed, subtriangular in lateral and mesal views, with one or two long distal setae; telopodite (**t**) smaller than mesal lobe, egg-shaped. Mesomere (Figs 4G, H, J, 5D, E, G, 6A) spoon-shaped in anterior view, slightly sigmoid in lateral and mesal views; anterodistal margin slightly denticulate; anterior side distally concave and microsquamose. Opisthomere (Figs 4G, H, 5D, E, 6A) straight and slender with distal half slightly directed anteriad; proximomesal spine present; anteromesal lamella poorly developed; velum (**v**) unipartite, tapering anterodistad, with fimbriated tip; solenomere (**s**) narrow, tubelike.

##### Habitat.

**Figure 7.**
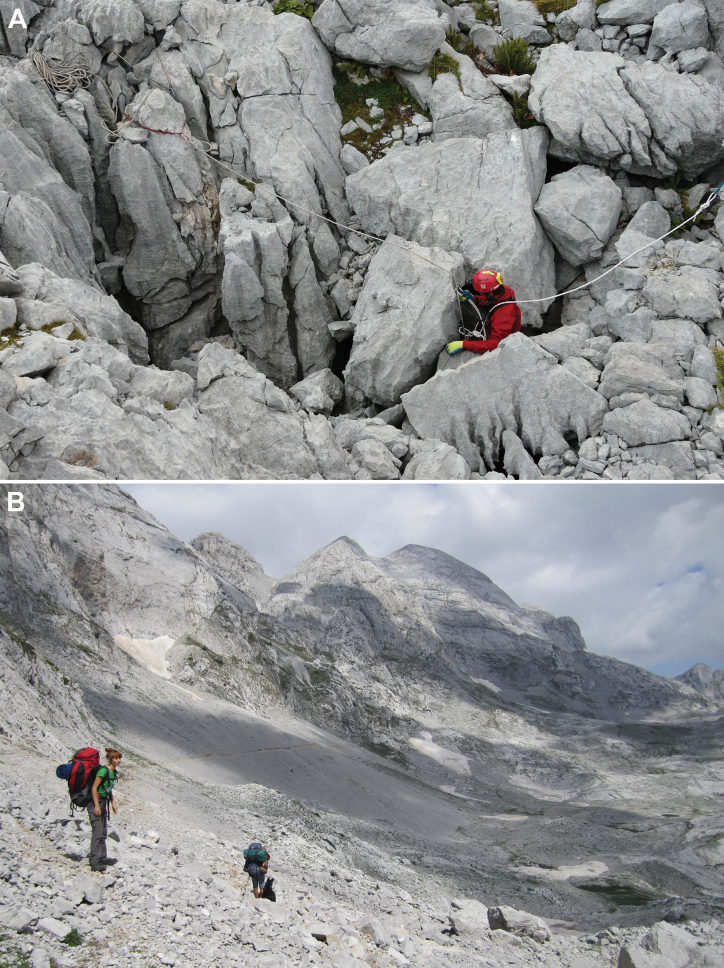
Ru Cave and surroundings **A** entrance to cave (photograph: Vladimir Georgiev) **B** Radohimës massif of the Prokletije Mountains where the cave is located (photograph: Marinko Malenica).

The Ru Cave (Fig. 7A) was discovered by Bulgarian speleologists in 2010. It is located at an altitude of 2225 m in the Radohimës massif (Fig. 7B) of the Prokletije Mountains. With an explored depth of 574 m, this cave (length 1300 m) is the second deepest cave known in Albania. It has a predominantly vertical character and is risky in terms of rockfall and flooding. The new species was found in a short horizontal meander whose bottom is covered by water, 460 m below the entrance. In addition to the new species, representatives of Lumbricidae, Leiodidae, and Opiliones, as well as Chordeumatida (probably *Macrochaetosoma* Absolon & Lang, 1933), were also found in the cave at various depths (Tamara Čuković and Vladimir Georgiev, pers. comm.).

##### Remarks.

Besides the chordeumatidan *Macrochaetosomatroglomontanum* Absolon & Lang, 1933 (see Absolon and Lang 1933; Stoev 2001), *Inversotyphlusammirandus* sp. nov. is the second undisputed troglobiotic millipede in the fauna of Albania. In addition, this is one of the rare cave-dwelling julids characterised by modifications of the body and mouthparts for a semiaquatic life and filtering diet, and also the first such millipede in the territory of Albania.

It is interesting to note that several ultraspecialized hygropetricolous leptodirinine beetles were recently described from this Albanian part of the Prokletiје Mountains (Giachino and Casale 2022).

In addition to the two unequivocal troglobionts mentioned above, there are two other Albanian millipede species that may potentially be troglobiotic, *Metonomastuspetrelensis* Mauriès, Golovatch & Stoev, 1997 and *Typhloiulusberoni* Mauriès, Golovatch & Stoev, 1997, both known from artificial galleries (Mauriès et al. 1997).

##### Distribution.

So far the new species is only known from a single pit in the Albanian part of the Prokletije Mountains.

###### Additional material examined

#### ﻿ Inversotyphlus edentulus

Taxon classificationAnimaliaJulidaJulidae

(Attems, 1951) comb. nov.

07651C6B-A868-59F5-854A-F8A8FB07DA0F

##### Material examined.

1 ♂ (IZB, Fig. 6C, see also Antić et al. (2018: 262, fig. 2B–F)), Montenegro, Cetinje, Njeguši, Njegoš Cave, 4 May 2013, T. Delić leg.

### ﻿Key to the species of the genus *Inversotyphlus* Strasser, 1962, stat. nov.

**Table:** 

1	Mouthparts modified for a hydrophilous lifestyle	**2**
–	Mouthparts normal	**3**
2	Head with two well developed labral lobes	***I.ammirandus* sp. nov.**
–	Head without labral lobes	***I.edentulus* (Attems, 1951), comb. nov.**
3	Mesomere higher than promere	**4**
–	Mesomere lower than promere	**6**
4	Promere slender in lateral and mesal views, clearly bent anteriad	***I.lobifer* (Attems, 1951), comb. nov.**
–	Promere robust, not or poorly bent anteriad	**5**
5	Distal part of promere slightly curved anteriad. Mesomere clavate, considerably higher than promere	***I.clavatus* (Antić, 2018), comb. nov.**
–	Distal part of promere not curved anteriad. Mesomere not clavate, only slightly higher than promere	***I.gellianae* (Makarov & Rađa, 2006), comb. nov.**
6	Opisthomere with a posterior node	***I.opisthonodus* (Antić, 2018), comb. nov.**
–	Opisthomere without posterior node	***I.gracilis* (Antić, 2018), comb. nov.**

To easily distinguish all seven species of the genus *Inversotyphlus* stat. nov., see also Fig. 6.

## ﻿Discussion

### ﻿Notes on the taxonomy of the Typhloiulini

In the time since Strasser’s (1962) remarkable work “Die Typhloiulini”, it appears that the tribe is one of the most studied julid groups in the Balkan Peninsula, especially in recent years when some more extensive studies have appeared (Vagalinski et al. 2015; Makarov et al. 2017; Antić et al. 2018). One of the reasons for this is the long tradition of speleobiological research in the Balkans. Representatives of this group are frequently collected from caves and endogean habitats in this region, and new material is often available for study. Nevertheless, the validity of this tribe is still questioned (Mauriès et al. 1997; Vagalinski et al. 2015; Tabacaru and Giurginca 2016; Antić et al. 2018). Not only does the tribe itself not seem to represent a natural unit, but the monophyly of the genus *Typhloiulus* is also questionable (Vagalinski et al. 2015). As mentioned earlier, the genus *Typhloiulus* comprises several subgenera, some of which are very doubtful, while some are well defined. Recently, Vagalinski et al. (2022) have shown that one of them, *Stygiiulus* Verhoeff, 1929 (see Verhoeff 1929), is actually a valid genus that does not even belong to the tribe Typhloiulini. In addition, the subgenus Alpityphlus Strasser, 1967 (see Strasser 1967) was synonymised with *Stygiiulus* by Vagalinski et al. (2022).

Another well-defined group is *Inversotyphlus* stat. nov. According to Antić et al. (2018), this lineage includes at least five or six species known so far exclusively from caves in the Adriatic parts of the central and southern Dinarides. In addition to *Typhloiuluslobifer* Attems, 1951 (Fig. 6G) and *T.gellianae* Makarov & Rađa, 2006 (Fig. 6E), as undoubted representatives of *Inversotyphlus* stat. nov., Antić et al. (2018) also included three newly described species, *T.clavatus* Antić, 2018 (Fig. 6B), *T.gracilis* Antić, 2018 (Fig. 6F), and *T.opisthonodus* Antić, 2018 (Fig. 6D). According to Antić et al. (2018), all five species share some similarities in habitus and gonopod structures, including a slender opisthomere with a characteristic velum ending with a thin process carrying a few fimbriae, presence of a poorly developed but noticeable anterior opisthomeral lamella, and the simple, more or less blunt apex of the solenomere. Previously, Makarov et al. (2017) used molecular and semiochemical data to show that the two species of *Inversotyphlus* stat. nov. studied grouped in a separate clade. Based on all this, Antić et al. (2018) speculated that *Inversotyphlus* stat. nov. may indeed be a natural group and probably deserves full generic status. In this paper, we took the opportunity to raise *Inversotyphlus* stat. nov. to the genus level.

Based on similarities in opisthomere configuration with members of the genus *Inversotyphlus* stat. nov., as well as geographical proximity, Antić et al. (2018) discussed *Typhloiulusedentulus* as another possible member of this group. Since *T.edentulus* possesses peculiar modifications (see below), Strasser (1962) established the subgenus Attemsotyphlus Strasser, 1962 to accommodate this species. The unusual modifications, also observed in *T.edentulus*, has occurred several times in different groups and have no phylogenetic significance (Enghoff 1985; Antić et al. 2017; Antić and Reip 2020). Here, we fully support the initial idea of Antić et al. (2018) that *T.edentulus* is closely related to members of *Inversotyphlus* stat. nov., and therefore we formally transfer that species to *Inversotyphlus* stat. nov. and propose *Attemsotyphlus* syn. nov. as a junior subjective synonym.

In our opinion, the new species fits well into the concept of *Inversotyphlus* stat. nov., both in the opisthomere configuration and in its geographical distribution. The new species is the southernmost known species of the group, occurring on the southern border of the Dinaric region. With this in mind, the genus *Inversotyphlus* stat. nov. should include at least seven species (Fig. 6)–*I.ammirandus* sp. nov., *I.clavatus* comb. nov., *I.edentulus* comb. nov., *I.gellianae* comb. nov., *I.gracilis* comb. nov., *I.lobifer* comb. nov. and *I.opisthonodus* comb. nov. *Typhloiulusbosniensis* Strasser, 1966 (see Strasser 1966) might be another member of this, as far as known, Dinaric lineage. Curiously enough, Popa et al. (2019) recorded the northernmost representative of *Inversotyphlus* stat. nov., *I.gellianae* comb. nov. (as *Typhloiulusgellianae*), from a cave in Greece, which is remote from the type and only locality of this species on the island of Ugljan in Croatia. The drawings of the gonopods of the specimen from the Greek cave (Popa et al. 2019: 72, fig. 2) provide enough evidence that promere and mesomere are considerably different compared to *I.gellianae* comb. nov. Thus, the record of this species from a Greek cave should be disregarded. The specimen depicted by Popa et al. (2019) shows an opisthomere characteristic of the genus *Inversotyphlus* stat. nov. and most likely belongs to an undescribed species. With this in mind, the range of the genus *Inversotyphlus* stat. nov. probably includes areas of further south in the Balkan Peninsula.

### ﻿Notes on the adaptive modifications of mouthparts in cave-dwelling julids

With relatively constant levels of humidity and temperature, cave ecosystems procure a stable environment for their fauna, whereas the low oxygen levels and scarcity of nutrients generally render them extremely harsh places to live. Many myriapod species have, however, managed to prevail under these severe underground conditions, and they have evolved a number of morphological, physiological, and behavioural traits. The morphological modifications help these animals adapt to subterranean life and are perhaps the easiest for biologists to assess. Among the classical and most observed traits of troglomorphism are, for example, the depigmentation of the cuticle, partial or complete reduction of visual perception, and elongation of appendages. Some millipedes are even amphibious, having a filtering diet due to modifications of their mouthparts, which is evident in the species described here. Among the most notable examples of troglomorphic adaptation, and similar to our new species, is found in *Leucogeorgiamystax* Antić & Reip, 2020, which was discovered in the Arabika Massif in the Caucasus; this species bears a bilobed labrum and gnathochilarium with long distal setae in transverse row and distolateral bundles appearing as “moustaches” (Antić and Reip 2020: 64, 65, figs 39C, D, 40A–G), which is very similar to *I.ammirandus* sp. nov. Besides *I.ammirandus* sp. nov., there are several other modified cave-dwelling julids which are characterised by modified mouthparts but also have a shorter body with fewer body rings in comparison with their “normal” congeners (Enghoff 1985; Antić et al. 2017, 2018; Antić and Reip 2020; Vagalinski et al. 2022).

As already stated, these modifications seem to be the result of convergent evolution and do not reflect any phylogenetic relationships. However, very little is known about the detailed morphology of these structures, and an in-depth study could clarify the exact function of these modifications as well as the biology of these fascinating creatures and their evolution.

## Supplementary Material

XML Treatment for Inversotyphlus

XML Treatment for Inversotyphlus ammirandus

XML Treatment for Inversotyphlus edentulus

## ﻿Additional information

### Author ORCIDs

Dragan Antić https://orcid.org/0000-0002-1231-4213

Nesrine Akkari https://orcid.org/0000-0001-5019-4833

### Data availability

All of the data that support the findings of this study are available in the main text.
